# Intracellular *Salmonella* delivery of an exogenous immunization antigen refocuses CD8 T cells against cancer cells, eliminates pancreatic tumors and forms antitumor immunity

**DOI:** 10.3389/fimmu.2023.1228532

**Published:** 2023-10-05

**Authors:** Vishnu Raman, Lars M. Howell, Shoshana M. K. Bloom, Christopher L. Hall, Victoria E. Wetherby, Lisa M. Minter, Ashish A. Kulkarni, Neil S. Forbes

**Affiliations:** ^1^ Department of Chemical Engineering, University of Massachusetts, Amherst, MA, United States; ^2^ Ernest Pharmaceuticals, LLC, Hadley, MA, United States; ^3^ Molecular and Cell Biology Program, University of Massachusetts, Amherst, MA, United States; ^4^ Department of Veterinary and Animal Sciences, University of Massachusetts, Amherst, MA, United States; ^5^ Institute for Applied Life Science, University of Massachusetts, Amherst, MA, United States

**Keywords:** bacterial therapy, CD8 T cells, intracellular antigen delivery, refocus of vaccine immunity, exogenous antigen, recall antigen, recall response, cancer immunotherapy

## Abstract

**Introduction:**

Immunotherapies have shown great promise, but are not effective for all tumors types and are effective in less than 3% of patients with pancreatic ductal adenocarcinomas (PDAC). To make an immune treatment that is effective for more cancer patients and those with PDAC specifically, we genetically engineered Salmonella to deliver exogenous antigens directly into the cytoplasm of tumor cells. We hypothesized that intracellular delivery of an exogenous immunization antigen would activate antigen-specific CD8 T cells and reduce tumors in immunized mice.

**Methods:**

To test this hypothesis, we administered intracellular delivering (ID) Salmonella that deliver ovalbumin as a model antigen into tumor-bearing, ovalbumin-vaccinated mice. ID Salmonella delivers antigens by autonomously lysing in cells after the induction of cell invasion.

**Results:**

We showed that the delivered ovalbumin disperses throughout the cytoplasm of cells in culture and in tumors. This delivery into the cytoplasm is essential for antigen cross-presentation. We showed that co-culture of ovalbumin-recipient cancer cells with ovalbumin-specific CD8 T cells triggered a cytotoxic T cell response. After the adoptive transfer of OT-I CD8 T cells, intracellular delivery of ovalbumin reduced tumor growth and eliminated tumors. This effect was dependent on the presence of the ovalbumin-specific T cells. Following vaccination with the exogenous antigen in mice, intracellular delivery of the antigen cleared 43% of established KPC pancreatic tumors, increased survival, and prevented tumor re-implantation.

**Discussion:**

This response in the immunosuppressive KPC model demonstrates the potential to treat tumors that do not respond to checkpoint inhibitors, and the response to re-challenge indicates that new immunity was established against intrinsic tumor antigens. In the clinic, ID Salmonella could be used to deliver a protein antigen from a childhood immunization to refocus pre-existing T cell immunity against tumors. As an off-the-shelf immunotherapy, this bacterial system has the potential to be effective in a broad range of cancer patients.

## Introduction

Immunotherapy has proven to be extremely effective for many, but not all tumors types (1–3). For example, in pancreatic ductal adenocarcinomas (PDAC), immune checkpoint inhibitors (ICIs) are effective in less than 3% of patients (4–7). Despite the limitation of ICIs, recent successes with chimeric antigen receptor (CAR) T cell therapy in individual patients (8–11), suggests that T cell therapies can be effective against PDAC. Alternate methods are needed to build upon this potential while avoiding the difficulty of scaling these treatments (12). A therapeutic strategy that directs pre-existing pools of T cells against tumors could provide a universal treatment for patients with PDAC and ICI-resistant tumors.

Delivering an antigen from a prior immunization into cancer cells would redirect CD8 T cells from a vaccine against the recipient cells. Delivery into the cytoplasm is a critical component of this technique because it is necessary to induce a cytotoxic T cell response (12, 13). Most protein delivery mechanisms (e.g. nanoparticles, cell-penetrating peptides, and antibody drug conjugates) deliver proteins to early and late endosomes, where they are trafficked to the lysosome and degraded (14–16). In contrast, proteins delivered to the cytoplasm would be processed by the proteasome and antigen-presented on the cell surface (12, 17–19) to interact with CD8 T cells (12, 20). In addition to the direct elimination of presenting cancer cells, recognition of foreign antigens by immune cells in tumors is a critical step that can lead to the acquisition of antitumor immunity (21–24).

**Figure 1:**
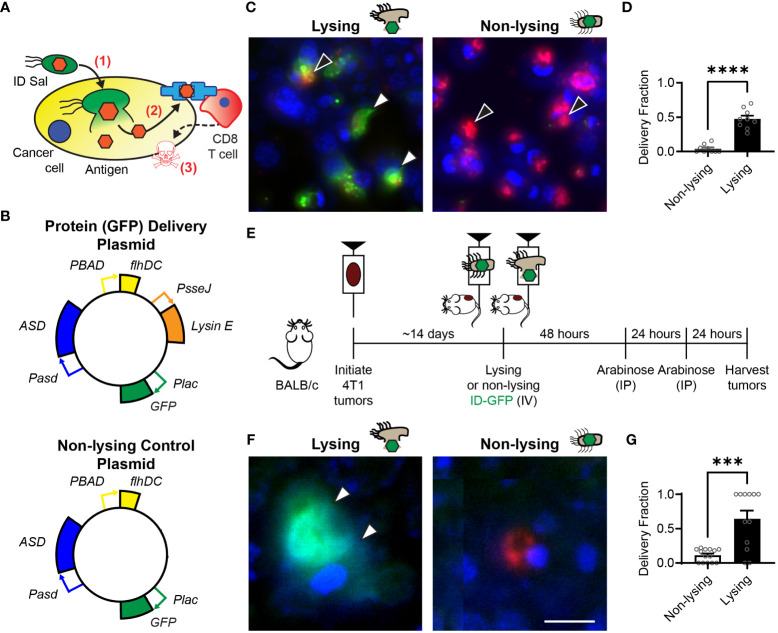
Intracellular delivering (ID) Salmonella deposit antigens into cells in tumors. **(A)** After ID Salmonella invade cancer cells (1), the bacteria autonomously lyse and deposit recombinant antigens into the cellular cytoplasm (2). Presentation of the delivered antigen on the cell surface activates antigen-specific CD8 T cells, which kill the presenting cancer cells (3). **(B)** Salmonella that intracellularly deliver GFP were created by transformation with a plasmid that contains circuits that produce the protein (*Plac-GFP*), control cell invasion (*PBAD-flhDC*), maintain the plasmid without antibiotics (*Pasd-ASD*), and cause lysis after cell invasion (*PsseJ-LysE*). Control Salmonella (*bottom*) were created that invade and produce GFP, but do not lyse. **(C)** Lysing (ID) and non-lysing control Salmonella were administered to 4T1 cancer cells in culture (*n* = 9). After cell invasion, GFP (*green*, *white arrows, left*) was released from intracellular ID Salmonella (red, black arrows, left), but was not released from non-lysing controls (red, black arrows, right). **(D)** ID Salmonella delivered GFP to significantly more cells than non-lysing controls (P < 0.0001). **(E)** Intracellular delivery was measured in BALB/c mice implanted with 4T1 tumor cells. Once tumors reached 500 mm^3^ (about 14 days), mice were intravenously injected with lysing (ID-GFP) or non-lysing Salmonella. After 48 and 72 h, *flhDC*-driven cell invasion was induced with IP injections of arabinose. At 96 h, tumors were harvested for histological examination. **(F)** ID-GFP Salmonella invaded and intracellularly delivered GFP throughout the cytoplasm of cells within tumors (white arrows, left). Non-lysing Salmonella (red) invaded cancer cells but did not deliver GFP (right). **(G)** Protein delivery was six times greater in cells containing ID-GFP Salmonella compared to non-lysing controls (P = 0.0001; *n* = 14 for non-lysing and *n* = 12 for lysing). Data are shown as means ± SEM. Statistical comparison is a two-tailed, unpaired Student’s t test with asterisks indicating significance (***, P < 0.001; ****, P < 0.0001). The scale bar in **(F)** is 10 µm.

We have recently created intracellular delivering (ID) Salmonella to release proteins into the cytoplasm of cancer cells (*step 1*, **Figure 1A**) (25). This delivery system utilizes innate Salmonella mechanisms (26, 27) to control invasion into cancer cells (25). After cell invasion, an engineered gene circuit triggers bacterial lysis and releases expressed proteins (25). The autonomous lysis system makes the therapy safe and non-toxic by clearing the bacteria after delivery of the protein payload (25). In addition to cytoplasmic delivery, ID Salmonella accumulate in tumors over healthy organs more than 3000-fold after intravenous injection (28, 29). There are five predominant mechanisms that lead to this accumulation: (1) increased blood flow following inflammation (29); (2) entrapment in the tumor vasculature (28); (3) chemotaxis into the tumor interstitium (30, 31); (4) preferential replication in the tumor microenvironment (30, 31); and (5) immune protection in the privileged tumor microenvironment (32). Other strategies have demonstrated the potential of microbial immunotherapies by showing that engineered bacteria can deliver tumor neoantigens (33) and checkpoint nanobodies (34) into tumors, while promoting T cell infiltration (35).

Here, we describe the creation of a bacterial immune therapy that uses ID Salmonella to deliver an exogenous immunization antigen into the cytoplasm of cancer cells (step 1, **Figure 1A**). Cytoplasmic antigens that are presented on the cell surface (step 2) are recognized by cytotoxic CD8 T cells (step 3) (17, 36). We hypothesized that delivering an exogenous antigen into cancer cells with ID Salmonella activates antigen-specific CD8 T cells, reduces tumor volume and increases survival in immunized mice. To test this hypothesis, we engineered ID Salmonella to deliver ovalbumin as a model of an antigen from a prior immunization. We used an *in vitro* cell invasion assay, T cell co-culture, and fixed-cell microscopy to quantify delivery into cancer cells and measure the CD8 T cell response. We used adoptive T cell transfer and immunization to quantify the primary effect of intracellular antigen delivery on tumor growth and survival. To explore the extent that this treatment forms antitumor immunity, we re-challenged mice with tumor cells after the primary tumors had cleared. We measured these immune responses in the highly immunosuppressive KPC tumor model that does not respond to ICIs (37, 38). Results from these experiments show that by refocusing pre-existing, T cell immunity against tumors, antigen delivery with ID Salmonella is an immunotherapy that could be effective for a wide range of cancer patients.

## Results

### Engineered *Salmonella* deliver exogenous antigens into cancer cells

Intracellular delivering (ID) Salmonella were created by transformation with a delivery platform that controls cell invasion, triggers intracellular lysis and delivers proteins into cancer cells (**Figure 1B**, top). This plasmid contains genetic circuits that (1) constitutively produce green fluorescent protein (GFP), *Plac-GFP*; (2) control cell invasion, *PBAD-flhDC*; (3) maintain plasmids after injection in mice, *Pasd-asd*; and (4) lyse the bacteria after cell invasion, *PsseJ-LysE*. A control strain was created by transforming bacteria with a plasmid that produces GFP (*Plac-GFP*) and controls invasion (*PBAD-flhDC*) but does not contain the genetic circuit for autonomous lysis (*PsseJ-LysE*; **Figure 1B**, bottom). When administered to 4T1 cancer cells, ID Salmonella delivered GFP into the cellular cytoplasm (**Figure 1C**, left). Non-lysing controls did not release any GFP (**Figure 1C**, right). Lysing Salmonella delivered GFP to significantly more cells than non-lysing controls (P < 0.0001; **Figure 1D**).

To measure the extent that the lysis system promotes protein delivery to cancer cells in tumors, ID-GFP Salmonella were administered to mice with 4T1 mammary tumors (**Figure 1E**). Control mice were administered parental Salmonella that do not lyse. Two days after bacterial injection, all mice were injected with arabinose to activate the *PBAD-flhDC* circuit and induce cell invasion (**Figure 1E**). In mice that received ID-GFP Salmonella, the cytosol of cancer cells was filled with bacterially produced GFP (**Figure 1F**, left). In control mice, cells contained Salmonella, but these intracellular bacteria did not release any GFP. (**Figure 1F**, right). ID-GFP Salmonella delivered protein to significantly more cells than control bacteria (P = 0.0001, **Figure 1G**). In cells with intracellular bacteria, ID-GFP Salmonella delivered GFP to more than 60% of cells (P = 0.0002, **Figure 1G**).

### Intracellular bacterial antigen delivery induced a cytotoxic CD8 T cell response

**Figure 2:**
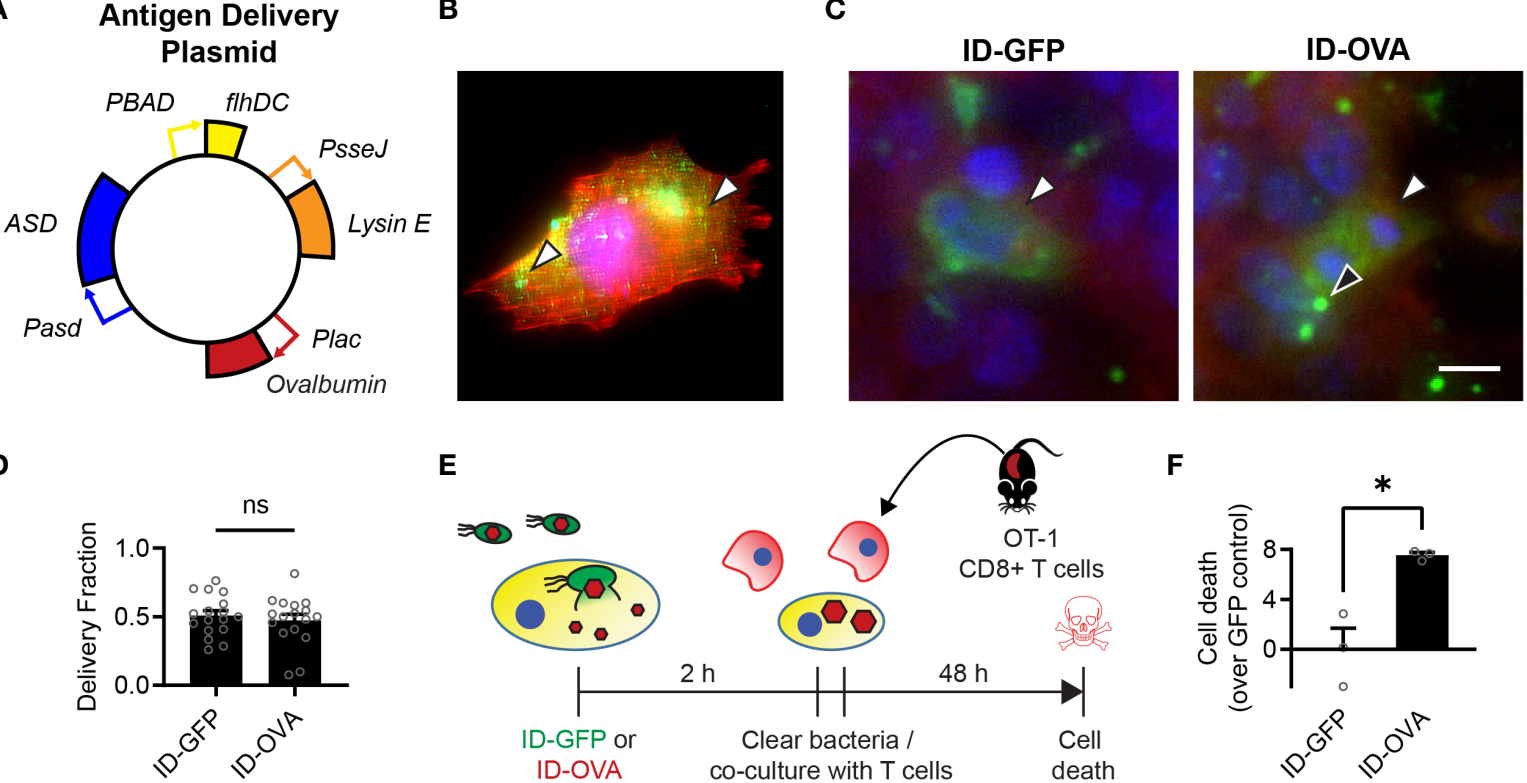
Bacterial delivery of ovalbumin induces a specific CD8 T cell response. **(A)** Salmonella with a genomic *flhD* and *ASD* double knockout were transformed with an antigen-delivery plasmid to create the ID-OVA strain. The plasmid contains four genetic circuits: (1) *PBAD-flhDC* to control cell invasion, (2) *Plac-OVA* to produce ovalbumin constitutively, (3) *PsseJ-LysE* to induce autonomous intracellular lysis, and (4) *Pasd-ASD* for plasmid retention. **(B)** After administration to 4T1 cancer cells, ID-OVA invaded the cells and delivered ovalbumin (*green, arrows*) throughout the cytoplasm. **(C)** ID-OVA and ID-GFP were administered to Hepa 1-6 cells at a multiplicity of infection (MOI) of 20 (*n* = 17). After cell invasion, ID-GFP and ID-OVA lysed and released their produced protein into the cellular cytoplasm (*green*, *white arrows*). Some intracellular bacteria did not lyse (*black arrow*). Both GFP and OVA were C-terminally myc tagged and identified with an anti-myc antibody. **(D)** There was no significant difference in the fraction of cells with delivered protein. **(E)** ID-OVA Salmonella were administered to Hepa 1-6 cancer cells to measure the effect of ovalbumin delivery on T cell cytotoxicity. ID-OVA were administered at a MOI of 10:1 for 2 h CD8 T cells were isolated from the spleens of OT-I mice and were activated with anti-CD3ζ antibody, followed by IL-2 and anti-CD28 antibody. Immediately after bacterial clearance with gentamicin, the isolated T cells were co-cultured with the cancer cells at a ratio of 10:1 for 48 h **(F)** The activated CD8 T cells killed more cancer cell after administration of ID-OVA compared to ID-GFP (*, P = 0.011; *n* = 3). Cell death was determined by release of Calcein AM from the cancer cells. Measurements are arbitrary units and were normalized by the ID-GFP controls, which indicate death due to cell culture and bacterial invasion. Data are shown as means ± SEM. The statistical comparisons in **(D, F)** are two-tailed, unpaired Student’s t tests. Asterisks indicate significance (*, P < 0.05). The scale bar in **(C)** is 10 µm.

To create the bacterial immunotherapy, we transformed Salmonella with a plasmid that encodes for the production and intracellular release of ovalbumin, as a model of an immunization antigen (**Figure 2A**). This engineered ID-OVA strain has the same circuits as ID-GFP to control invasion and lysis. When administered to 4T1 cancer cells, ID-OVA lysed and delivered ovalbumin that diffused throughout the cytosol (**Figure 2B**). Administration of either ID-GFP or ID-OVA equally delivered proteins into approximately 50% of cells (**Figures 2C, D**).

To measure the effect of ovalbumin delivery on T cell cytotoxicity, ID-OVA Salmonella were administered to Hepa 1-6 cancer cells for 2 hours (**Figure 2E**). The response was compared to administration of ID-GFP as a control. After removal of extracellular bacteria, activated OT-I CD8 T cells were immediately added to the cultures for 48 hours at a ratio of ten CD8 T cells to one cancer cell. In these co-cultures, the CD8 T cells killed more cancer cells after administration of ID-OVA compared to control ID-GFP Salmonella (P < 0.05, **Figure 2F**).

### Exogenous antigen delivery to tumors induced an antigen-specific T cell response

**Figure 3:**
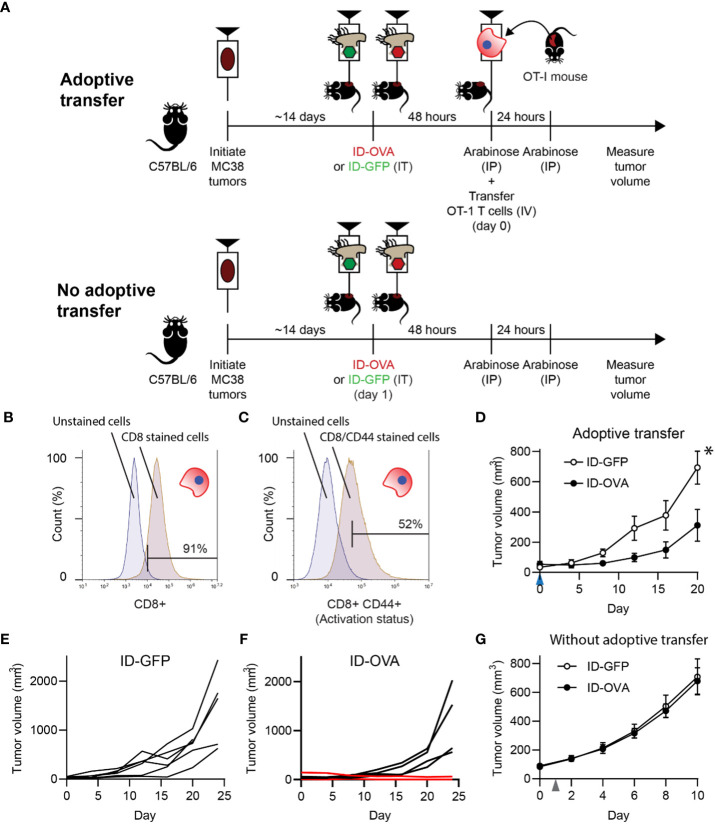
Bacterial delivery of ovalbumin induced an antigen-specific T cell response. **(A)** To determine the effect of antigen delivery on tumor volume, ID-OVA Salmonella were administered to mice with adoptively transferred CD8 T cells from OT-I mice. To determine the dependence on T cells, bacteria were also administered to control mice that did not receive transferred T cells. For all treatment groups, MC38 tumor cells were injected into wild-type C57BL/6 mice. When tumors reached approximately 50 mm^3^, they were injected with either ID-GFP or ID-OVA. Two days after bacterial injection (on day 0), OT-I T cells were intravenously injected into the adoptive transfer mice and tumor volumes were recorded twice a week. Arabinose (100 mg) was injected IP at 48 and 72 hours after bacterial injection to induce *flhDC* expression. **(B, C)** The purity and activation of isolated OT-I T cells was determined by expression of CD8 **(B)** and co-expression of CD8 and CD44 **(*C*)**. The left peaks are unstained cells (fluorescence minus one controls) that define the upper bounds for the background signal. **(D)** Mice with adoptively transferred OT-I CD8 T cells and administered ID-OVA had reduced tumor growth compared to mice administered ID-GFP (P = 0.031 at 20 days; *n* = 6). OT-I T cells were transferred on day 0 (*blue arrow*). Volume is plotted to day 20, when one mouse was removed from the study. **(E)** Individual tumor growth trajectories of mice administered with ID-GFP. **(F)** Individual tumor growth trajectories of mice administered with ID-OVA. One mouse had a partial response (*lower red line*) and another had a complete response (*upper red line*). The tumor in the mouse with the complete response shrank over the experiment and was undetectable by day 28. The tumor in the mouse with the partial response was undetectable for much of the experiment but started to grow (0.5 mm^3^) on day 24. **(G)** In mice without adoptive transfer, there was no difference in tumor response to ID-OVA and ID-GFP (*n* = 8). Bacteria were injected on day 1 (*grey arrow*). Data are shown as means ± SEM. Statistical comparison in **(D)** is a two-tailed, unpaired Student’s t tests with asterisk indicating significance (*, P < 0.05).

To test if exogenous protein delivery could induce an antigen-specific T cell response, ID-OVA Salmonella were administered to mice with MC38 tumors (**Figure 3A**). Five days after intratumoral injection of either ID-OVA or control ID-GFP, half of the mice were injected with activated, ovalbumin-specific CD8 T cells (**Figure 3A**). No T cells were transferred into the remaining mice (**Figure 3A**). The injected OT-I T cells were 91% pure (**Figure 3B**) and over half expressed high levels of the activation marker, CD44 (**Figure 3C**). Mice treated with ID-OVA had significantly reduced tumor growth compared with mice treated with ID-GFP controls (P < 0.05; **Figure 3D**). None of the six mice treated with ID-GFP responded to bacterial injection (**Figure 3E**). In the ID-OVA group, one mouse had a partial response, and another had a complete response (red lines, **Figure 3F**). In the groups without adoptive transfer, there was no difference in tumor response between mice that received ID-OVA and ID-GFP (**Figure 3G**), indicating that the tumor response was mediated by the OT-I CD8 T cells.

### Refocusing vaccine immunity against tumors with bacterial antigen delivery

**Figure 4:**
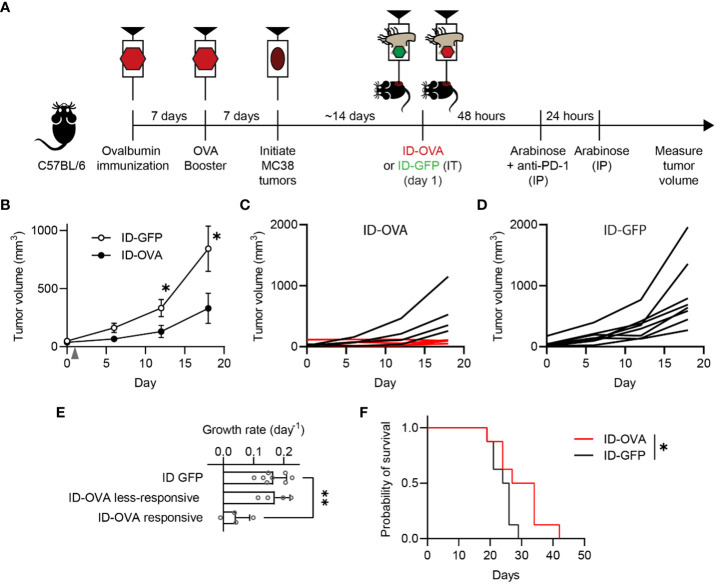
Exogenous antigen delivery with ID Salmonella refocuses vaccine immunity against tumors. **(A)** C57BL/6 mice were immunized against ovalbumin with two intraperitoneal injections of ovalbumin and poly(I:C), as an adjuvant, spaced seven days apart. Seven days after the second ovalbumin injection, the immunized mice were subcutaneously injected with 1×10^5^ MC38 tumor cells. Once tumors were between 50-75 mm^3^ (about two weeks), the mice were intratumorally injected with either ID-GFP or ID-OVA (on day 1). The mice also received intraperitoneal injections of 50 µg of anti-PD-1 checkpoint blockade 48 h after bacterial injection. **(B)** Ovalbumin immunized mice administered with ID-OVA had significantly slower tumor growth compared to control ID-GFP mice (P = 0.044 at 12 days and P = 0.049 at 18 days; *n* = 8). Bacteria were injected on day 1 (*grey arrow*). **(C)** By 18 days after bacterial administration, four of the eight mice administered ID-OVA had tumor volumes less than 110 mm^3^ (*red lines*). **(D)** Comparatively, at the same time point, none of the mice injected with ID-GFP had tumors less than 250 mm^3^. **(E)** The growth rate of responsive ID-OVA tumors was significantly lower than ID-GFP tumors (P = 0.0012; *n* = 8 for ID-GFP and *n* = 4 for responsive and less-responsive ID-OVA). **(F)** Administration of ID-OVA to ovalbumin-immunized mice significantly increased survival compared to control ID-GFP mice (*, P = 0.0480). Data are shown as means ± SEM. The statistical comparisons in **(B, E, F)** are two-tailed, unpaired Student’s t tests; ANOVA followed by Dunnett’s method; and a log-rank test, respectively. Asterisks indicate significance (*, P < 0.05; **, P < 0.01).

To test whether pre-existing, vaccine-generated immunity could be retargeted against cancer, antigen-delivering ID Salmonella were administered to vaccinated, tumor-bearing mice (**Figure 4A**). To establish immunity to an exogenous antigen, mice were vaccinated with two doses of ovalbumin and poly(I:C), which is a T_h_1 adjuvant that activates CD8 T cells against antigens (ovalbumin) in immunizations (**Figure 4A**). One week after the second vaccine dose, MC38 tumors were implanted in the mice. When the tumors formed, the mice were intratumorally injected with 2×10^7^ CFU (colony forming units) of either ID-OVA or control ID-GFP (**Figure 4A**). Tumor growth in mice injected with ID-OVA Salmonella was significantly reduced compared to mice injected with control ID-GFP (P < 0.05; **Figure 4B**). Four of the eight mice injected with ID-OVA had no significant tumor growth over eighteen days of observation (**Figure 4C**). In comparison, all tumors grew in control ID-GFP mice over the same period (**Figure 4D**). The growth rate of responsive ID-OVA tumors was 25% of ID-GFP tumors (P = 0.0012, **Figure 4E**). Mice administered with ID-OVA had prolonged survival compared to mice injected with ID-GFP (P = 0.0480, **Figure 4E**).

### Bacterial delivery of a vaccine antigen cleared pancreatic tumors and prevented tumor re-challenge

**Figure 5:**
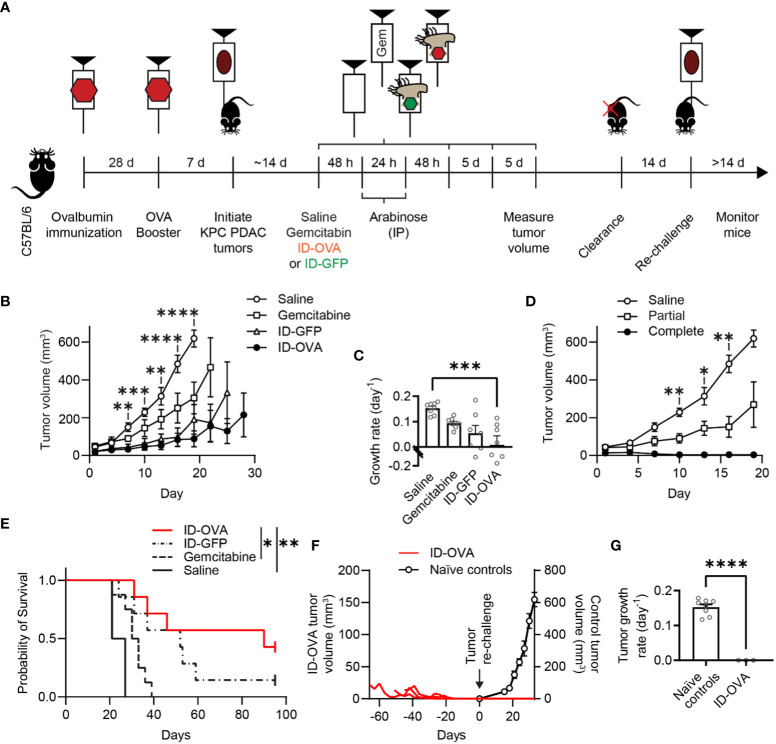
ID-OVA cleared KPC pancreatic tumors and prevented tumor re-challenge. **(A)** C57BL/6 mice were immunized with two intraperitoneal injections of ovalbumin and poly(I:C) spaced 28 days apart. Poly(I:C) was used to activate CD8 T cells against the ovalbumin in the immunizations. Pancreatic tumors were initiated seven days after the second immunization with a subcutaneous injection of 2×10^5^ KPC PDAC cells. Once tumors were between 30-50 mm^3^, they were injected with (1) saline (*n* = 8), (2) 50 mg/kg of gemcitabine (*n* = 8), (3) 2×10^7^ CFU control ID-GFP Salmonella (*n* = 7), or (4) 2×10^7^ CFU of ID-OVA Salmonella (*n* = 7). These injections continued every five days until mice were removed from the study or tumors were too small to be detected (four injections for all mice). All mice received intraperitoneal injections of 400 mg of arabinose 48 and 72 hours after therapeutic administration. After treatment, tumor volume was measured every three days. Mice with completely cleared primary tumors were re-challenged with 1x10^5^ KPC PDAC cells on the opposite flank 14 days after clearance and monitored for tumor regrowth for at least 14 days. **(B)** Tumor volume as a function of time. From day 7 to 19, tumors from mice injected with ID-OVA were significantly smaller than saline controls (d 7, P = 0.0052; d 10, P = 0.00016; d 13, P = 0.0031; d 16, P < 0.0001; d 19, P < 0.0001). **(C)** Treatment with ID-OVA significantly reduced the growth rate of KPC PDAC tumors (P = 0.0004). **(D)** Three mice treated with ID-OVA had complete responses and the remaining four had partial responses. Between days 10 and 16, the tumors in mice with partial responses were significantly smaller than saline controls (d 10, P = 0.0075; d 13, P = 0.036; d 16, P = 0.0046). **(E)** Treatment with ID-OVA increased survival compared to saline (P = 0.0012) and gemcitabine (P = 0.026). **(F)** After treatment with ID-OVA, the volume of tumors (*red lines*) of three mice completely cleared (*left axis*). Two weeks after clearance, mice were injected with KPC PDAC cells on the opposite flank. No new tumors appeared. For comparison, tumor volumes of naïve controls injected with KPC PDAC cells (*right axis*) are shown, aligned at the same injection time. **(G)** The growth rates of re-implanted tumors were significantly less than naïve controls (P < 0.0001). Data are shown as means ± SEM. Statistical comparisons in **(B, D)** are ANOVA with Bonferroni correction; in **(C)** are ANOVA followed by Dunnett’s multiple comparisons test; in **(E)** are log-rank tests with Bonferroni correction; and in **(G)** are two-tailed, unpaired Student’s t tests. Asterisks indicate significance (*, P < 0.05; **, P < 0.01; ***, P < 0.001; ****, P < 0.0001).

To test its efficacy against pancreatic cancer, ID-OVA was administered to immunocompetent C57BL/6 mice with KPC tumors (**Figure 5**). The KPC tumor model is driven by *KRAS* and p53 mutations that are common in human pancreatic cancer (37). The tumors have highly immunosuppressive microenvironments and do not respond to ICIs (37, 38). Four groups of mice were all immunized with two doses of ovalbumin and poly(I:C) (**Figure 5A**). Poly(I:C) was used to activate CD8 T cells against the ovalbumin in the immunizations. Seven days after this immunization regimen, the mice were implanted with KPC pancreatic ductal adenocarcinoma (PDAC) tumors on the flank. After tumors formed, the mice were injected with one of four treatments (1) saline, (2) gemcitabine, (3) control ID-GFP Salmonella, or (4) ID-OVA Salmonella. Gemcitabine is a standard therapy for pancreatic cancer. All groups of mice were immunized against ovalbumin, but ovalbumin was only delivered to the ID-OVA group. Tumor clearance was monitored for 14 days, after which some mice were re-challenged with KPC PDAC cells on the opposite flank (**Figure 5A**).

ID-OVA significantly reduced tumor volume compared to saline controls (**Figure 5B**). On day 19, the average tumor in ID-OVA-treated mice was 14% of saline-treated mice (P < 0.0001). The difference between ID-OVA treatment and saline controls shows that the poly(I:C) adjuvant did not induce a significant antitumor response. Treatment with ID-OVA significantly reduced the growth rate of KPC PDAC tumors (P = 0.0004, **Figure 5C**). Of the mice treated with ID-OVA, three had a complete response and four had partial responses (**Figure 5D**). Between days 10 and 16, the average tumor size in mice with partial responses to ID-OVA was 49% of saline-treated controls (P = 0.0046 on d 16; **Figure 5D**).

Treatment with ID-OVA antigen-delivering bacteria increased mouse survival and prevented tumor re-implantation (**Figures 5E–G**). In these mice with KPC PDAC tumors, ID-OVA significantly increased survival compared to both saline (P = 0.0012) and gemcitabine (P = 0.026). The median survival after treatment with ID-OVA was 90 days compared to 31.5 and 52 days for gemcitabine and ID-GFP. In three of the treated mice, ID-OVA eliminated tumors by days 31, 46 and 52 (**Figure 5F**). Two weeks after tumor clearance, these three mice were re-challenged with KPC PDAC cells in the opposite flank and monitored for at least four weeks. No tumors formed in any of the mice (**Figure 5F**). For comparison, naïve tumors grew at a rate of 0.14 d^-1^ (P < 0.0001, **Figure 5G**). These results show that bacterial delivery of an immunization antigen induces a durable response that prevents the establishment of new tumors.

## Discussion

These results show that intracellular delivery of an immunization antigen with engineered Salmonella induces T cell cytotoxicity and eliminates tumors. When Salmonella delivered exogenous antigens into the cytoplasm of cancer cells in tumors, the peptides dispersed throughout the cytoplasm (**Figures 1**, **2**). Bacterial delivery of ovalbumin marked cancer cells as immunological targets to be cleared by CD8 T cells (**Figure 2**). In mice, intracellular delivery of ovalbumin reduced the volume of colon and pancreatic tumors (**Figures 3**–**5**). The dependence on adoptive transfer suggests that the tumor response was mediated by the CD8 T cells (**Figure 3**). In tumors, the induced T cell-cytotoxicity (**Figures 3**–**5**) matched the cytotoxicity observed in culture (**Figure 2**). Bacterial delivery of ovalbumin to immunized mice reduced tumor volume and increased survival (**Figure 4**), suggesting that intracellular antigen delivery redirects vaccine immunity to tumors. Coupling vaccination with intracellular antigen delivery eliminated pancreatic tumors and prevented tumor re-implantation (**Figure 5**). Efficacy in the immunosuppressive KPC model demonstrates the clinical potential of the approach to overcome immune resistance in PDAC.

**Figure 6:**
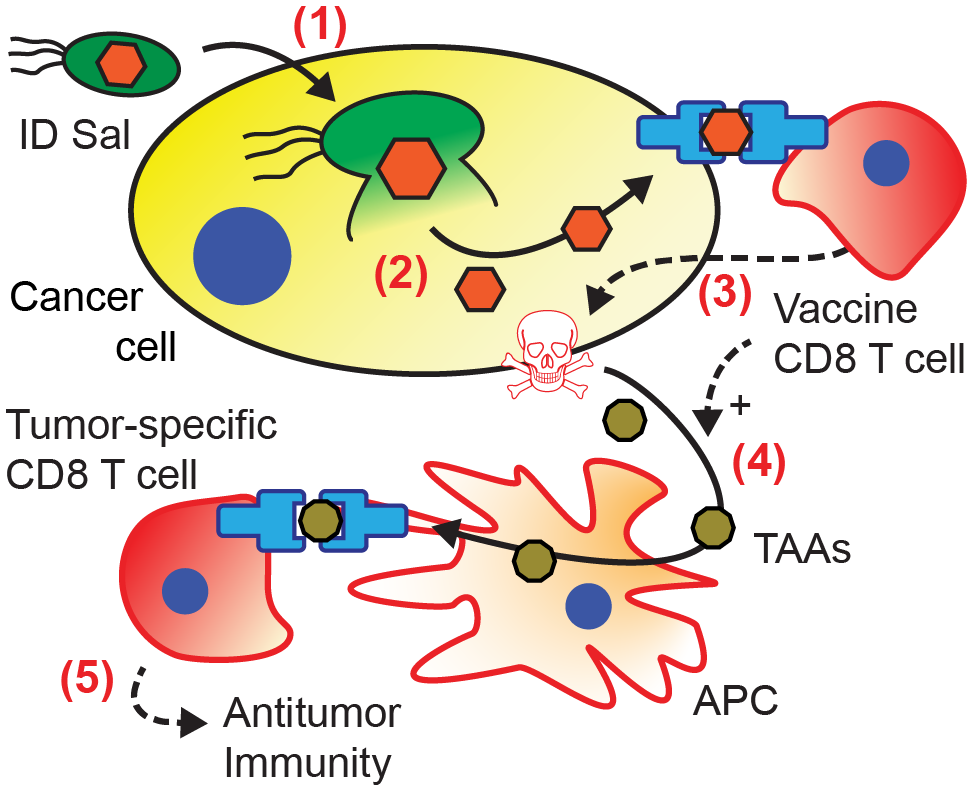
Mechanism of acquired antitumor immunity from intracellular bacterial antigen delivery. (1) Salmonella invade into cancer cells, and (2) autonomously lyse releasing bacterially expressed antigens (*orange*) into the cytoplasm. (3) Presentation of the delivered antigen activates antigen-specific vaccine CD8 T cells (12, 20), which kill the presenting cancer cells (36, 39–42). (4) Cancer cell death and T cell activation induce antigen presenting cells (APCs) to cross-present tumor associated antigens (TAAs, *brown*) (43–46). (5) Activation of tumor-specific CD8 T cells (23, 24, 47–49) leads to the formation of antitumor immunity (50–53).

The prevention of tumor re-challenge suggests that bacterial antigen delivery triggers the formation of antitumor immunity (**Figure 6**). In this mechanism, recognition of the vaccine antigen on the surface of cancer cell initiates an antigen cascade that leads to the formation of immunity against intrinsic tumor antigens (21–24). When co-cultured with cancer cells, ovalbumin-specific OT-I CD8 T cells preferentially killed cancer cells with bacterially delivered ovalbumin (**Figure 2F**). This specificity suggests that T cells recognized the ovalbumin antigen presented on the surface of the cancer cells (steps 1-3 in **Figure 6**). In mice, the dependence on transferred CD8 T cells (**Figure 3G**) indicates that T cell-mediated cytotoxicity is an essential component of the tumor response. In vaccinated mice, the tumor response was greater when the delivered antigen matched the vaccine antigen (**Figure 4**), suggesting that the vaccine T cells specifically recognized the delivered antigen. The development of the antitumor immunity (**Figure 5**) suggests that CD8 T cells played a critical role in the tumor response (54). The resistance to re-implantation of tumor cells, which did not contain ovalbumin (**Figure 5**), suggests that the developed immunity was to intrinsic tumor antigens (steps 4-5 in **Figure 6**).

This mechanism (**Figure 6**) is dependent on intracellular delivery of OVA. This delivery could not be confirmed in the mice that responded to the bacterial therapy because the tumors were eliminated (**Figures 3**–**5**). Using immunohistochemistry and microscopy, we previously showed that the ID Salmonella strain delivers proteins into the cytoplasm of cancer cells in culture and in mice (25). The specific response to OVA delivery over bacterial controls (**Figure 4**) provides additional evidence that OVA was delivered to the cellular cytoplasm. Although this mechanism is dependent on immune cells (e.g. dendric cells and CD8 T cells), it is not dependent on increased infiltration. While multiple bacterial mechanisms increase immune cell infiltration (55), the proposed mechanism is only dependent on the presence of T cells that respond to the presentation of ovalbumin. As a proof-of-principle study, we used intratumoral injections to reduce variability. Ultimately, this strategy would use intravenous bacterial injections. Our group and others have demonstrated that intravenously injected Salmonella specifically accumulates in tumors and delivers proteins into the cytoplasm of cancer cells once there (25, 28, 56–58).

The delivery of immunogenic antigens to tumors with Salmonella most likely induced a CD4 T cell response. Many groups have shown that Salmonella colonization in tumors activates CD4 T cells and induces the production of T_h_1 cytokines (33, 59–62). Infiltration of CD4 T cells is required for activation of CD8 T cells (63–65) and the tumor responses seen here (**Figures 3**–**5**). The T_h_1 cytokines produced by CD4 cells induce antigen-presenting cells (APCs) to cross-present tumor associated antigens (43–46) and are critical factors in the acquisition of antitumor immunity (**Figure 6**).

Immunization with the antigen prior to bacterial delivery is necessary because of the time required to form immunity. It is possible that OVA presentation after Salmonella delivery could have formed memory CD8 T cells (66). However, we did not see a tumor response after administering ID-OVA Salmonella to non-immunized mice that did not receive adoptively transferred CD8 T cells (**Figure 3G**). A likely reason for this lack of response is the time required (typically 4-8 days) to form memory CD8 T cells to a novel antigen (67). In addition, the memory CD8 T cell response could have been stronger after immunization because of T_h_1 adjuvant in the vaccine.

ID Salmonella is not the only bacterial delivery system that could deliver exogenous antigens to tumors. Previously, it has been shown that proteins can be delivered to tumors using the type three secretion system (T3SS) of *Yersinia enterocolitica* (68). Similar to ID Salmonella (**Figure 1**), T3SS delivery deposits recombinant proteins in the cytoplasm of cancer cells and could effectively deliver an exogenous antigen. Because the antigen must be unfolded and re-folded to pass through the T3SS needle (68), it is not effective at delivering all proteins (69, 70). In addition, the inducible *flhDC* circuit in ID Salmonella enables external control of cell invasion and ensures that delivery only occurs within tumors after bacteria have cleared from healthy tissue (25).

In the clinic, Salmonella*-*based antigen delivery could provide comprehensive, off-the-shelf immunotherapy. By utilizing established immunity to vaccine proteins, specific tumor antigens would not need to be identified, and the therapy could be effective against many tumors without modification. Rather than a model antigen, this bacterial system could deliver a protein antigen from a childhood vaccine to refocus the pre-existing vaccine immunity towards tumors. A single bacterial strain could be used for many patients, as long as the associated vaccine was widely administered across the population. Most (90.8%) adults in the United States have received immunizations that form memory CD8 T cells against multiple viral antigens (25–27). Without the need for tumor-specific antigenic profiling, antigen-delivering bacteria could prevent the formation of new tumors and metastases, similar to the re-challenge response observed in mice (**Figure 5**).

To make this strategy broadly effective in the clinic, it could be used with multiple vaccine antigens. This is possible because of the large genetic capacity of engineered bacteria to express multiple recombinant proteins. The average person has been administered nine different vaccines by three years of age (71). Engineered Salmonella could be designed to deliver a combinatorial range of vaccine-derived proteins to take advantage of this breadth of intrinsic immunity. Delivering multiple antigens would increase the probability that vaccine-associated T cells would infiltrate and activate within tumor tissue. An additional strategy that would increase efficacy would be delivery of booster vaccines to patients prior to bacterial antigen delivery. An antigen-specific booster would increase the number of vaccine-specific T cells in circulation and, therefore, the likelihood that vaccine T cells efficiently destroy cancer cells that present the exogenous vaccine antigen.

This study is the first to demonstrate that Salmonella can be used to repurpose immunization-derived immune cells to target tumors. A bacterial approach could provide new therapeutic options for patients with late-stage pancreatic cancer or patients with immunosuppressive tumors that do not respond to checkpoint inhibitors. It would be widely applicable to most patients with pre-existing immunity to vaccine antigens and would be less dependent on tumor subtype. Because the engineered Salmonella only lyse inside cells in tumors (25), the delivered antigen would be shielded from immunological detection and premature clearance in the blood. This therapy would be particularly beneficial if it increased recognition of tumor antigens and formed antitumor immunity, as suggested by the tumor re-challenge results. Redirecting pre-existing immune cells to fight cancer with tumor-selective Salmonella could serve as a rapidly deployable therapy that would be effective for many patients.

## Methods

### Plasmid design and strains

The protein delivery plasmid contains four gene circuits that activate intracellular lysis (*PsseJ-LysE*), control invasion (*PBAD-flhDC*), express GFP (*Plac-GFP-myc*), and maintain copy number (*Pasd-ASD*). The non-lysing control plasmid does not contain the intracellular lysing (*PsseJ-LysE*) circuit. The *myc* tag was added to the GFP to facilitate detection. Both of these plasmids contain the ColE1 origin and ampicillin resistance, and their creation is described previously (25). To create the ovalbumin delivery plasmid, the *ova* gene was amplified from plasmid #64599 (*Addgene*) using primers CCGCATAGTTAAGCCAGTATACATTTACACTTTATGCTTCCGGCTCGTATAATAAAAAAAAAAAAAAGGAGGAAAAAAAATGGGCTCCATCGGTGCAG and CTACAGATCCTCTTCTGAGATGAGTTTTTGTTCAGGGGAAACACATCTGCCAAA. The delivery plasmid was amplified using primers TCATCTCAGAAGAGGATCTGTAACTCCGCTATCGCTACGTGA and TGTATACTGGCTTAACTATGCGG. This PCR amplification preserved all genes within the plasmid and exchanged the *Plac-GFP-myc* genetic circuit for *Plac-ova-myc*. These plasmids were transformed into the *ΔflhD, Δasd* strain of VNP20009 as described previously (25) to generate ID-GFP and ID-OVA Salmonella. To detect antigen expression, ID-OVA was suspended in Laemmli buffer and *myc*-tagged ovalbumin was identified by immunoblot with rat anti-*myc* antibody (*Chromotek*).

### Cell culture

Four cancer cell lines were used in this study: 4T1 murine breast carcinoma cells, MC38 murine colon cancer cells, Hepa 1-6 murine hepatocellular carcinoma cells, and KPC PDA murine pancreatic cancer cells (*ATCC*, Manassas, VA). KPC (*LSL-Kras^G12D/+^;LSL-Trp53^R172H/+^;Pdx-1-Cre)* PDA and 4T1 cells were grown and maintained in Dulbecco’s Minimal Eagle Medium (DMEM) containing 3.7 g/L sodium bicarbonate and 10% fetal bovine serum. MC38 cancer cells were grown in RPMI-1640 supplemented with 2 g/L sodium bicarbonate, 10% fetal bovine serum and penicillin/streptomycin. For microscopy studies, 4T1 cancer cells were incubated in DMEM with 20 mM HEPES buffering agent and 10% FBS.

### Microscopy

Samples were imaged on a Zeiss Axio Observer Z.1 microscope. Fixed cells on coverslips were imaged with a 100x oil immersion objective (1.4 NA). Tumor sections were imaged with 20x objectives (0.3 and 0.4 NA, respectively). Fluorescence images were acquired with either 480/525 or 525/590 excitation/emission filters. All images were background subtracted and contrast was uniformly enhanced.

### Immunocytochemistry to detect protein delivery in cancer cells

To visualize and measure protein delivery, ID Salmonella were administered to cancer cells grown on glass coverslips. To prepare the coverslips, they were placed in 12-well plates and sterilized with UV light in a biosafety hood for 20 minutes. Cancer cells (either 4T1 or Hepa 1-6 cells) were seeded on the coverslips at 40% confluency and incubated overnight in DMEM. Concurrently, Salmonella were grown to an optical density (OD; at 600 nm) of 0.8. After incubation, the Salmonella were added to the cancer cell cultures and allowed to infect the cells for two hours. After this invasion period, the cultures were washed five times with 1 ml of phosphate buffered saline (PBS) and resuspended in 2 ml of DMEM with 20 mM HEPES, 10% FBS and 50 µg/ml gentamycin. The added gentamycin removes extracellular bacteria. After twenty-four hours of incubation, the media was removed and the coverslips were fixed with 10% formalin in PBS for 10 minutes. After fixing, the coverslips were blocked with intracellular staining buffer (ISB; phosphate-buffered saline [PBS] with 0.1% Tween 20, 1 mM EDTA, and 2% bovine serum albumin [BSA]) for 30 minutes. The Tween 20 in this buffer selectively permeabilizes mammalian cell membranes, while leaving bacterial membranes intact, as previously described (25). After permeabilization, coverslips were stained to identify Salmonella and delivered protein. Stained coverslips were washed three times with ISB and mounted to glass slides using 20 µl mountant with DAPI (ProLong Gold Antifade Mountant, *ThermoFisher*). Mounted coverslips were cured overnight at room temperature. Coverslips were imaged as described in the *microscopy* section.

### Measurement of delivery fraction

ID Salmonella was administered to cancer cells to measure the fraction of cells with delivered protein. Two experiments were used to measure (1) the necessity of the lysis gene circuit, and (2) the efficacy of delivering ovalbumin. The necessity of the *PsseJ-LysE* was measured by growing ID-GFP and non-lysing ID-GFP to an OD of 0.8 and infecting 4T1 cells at a multiplicity of infection (MOI) of 10 for two hours. The delivery of ovalbumin was measured by growing ID-OVA and ID-GFP to an OD of 0.8 and infecting Hepa 1-6 cells at an MOI of 20 for two hours. For both experiments, the bacteria were induced with 20 mM arabinose during co-infection. To eliminate extracellular bacteria after infection, the cells were washed five times with PBS and fresh media containing 50 µg/ml of gentamycin was added. After 24 hours of incubation, the coverslips were fixed and incubated in ISB for 30 minutes. Cells were stained to identify Salmonella with FITC-anti-Salmonella antibody (*Abcam*; 1:200 dilution) and GFP-myc, or OVA-myc with an anti-myc antibody (9E1, *Chromotek*; 1:200 dilution) for one hour at room temperature in a humidified chamber. Coverslips were incubated with secondary antibody (anti-rat alexa-568 antibody; 1:200 dilution) for one hour at room temperature.

Delivery fraction was quantified on a per-cell basis by assessing if cells were invaded with bacteria and contained delivered protein. Invaded cells were identified as nuclei bordering intracellular Salmonella. Cells with delivered protein stained for GFP throughout the cytosol. Delivery fraction was the number of cells with cytosolic protein delivery divided by the total number of infected cells. Image analysis was blinded and conducted without knowledge of the treatment group.

### Imaging ovalbumin delivery

Detailed images of delivered ovalbumin were obtained using the immunocytochemistry technique described above. ID-OVA was grown to an optical density of 0.8 and added to cultures of 4T1 cells at an multiplicity of infection (MOI) of 10 for two hours. After infection, the cells were washed and 50 µg/ml of gentamycin was added. After 24 hours of incubation, the coverslips were fixed and stained to identify OVA-myc with anti-myc antibody (9E1, *Chromotek*; 1:200 dilution). After primary staining, coverslips were incubated with secondary antibody (anti-rat alexa-488 antibody; 1:200 dilution) and Alexaflor-568-conjugated phalloidin (*ThermoFisher*; 1:200 dilution) to identify f-actin.

### Immunohistochemical detection of GFP delivery *in vivo*

To identify and quantify GFP delivery to tumor cells, two groups BALB/c mice with 4T1 tumors were injected with 2×10^6^ CFU of either ID-GFP or non-lysing ID-GFP Salmonella. Both groups of mice were injected (IP) with arabinose at 48 and 72 h post bacterial injection to induce *flhDC* expression. Ninety-six hours after bacterial injection, mice were sacrificed and tumors were excised.

Tumor sections were fixed in 10% formalin for 3 days. Fixed tumor samples were stored in 70% ethanol for 1 week. Tumor samples were embedded in paraffin and sectioned into 5 µm sections. Deparaffinization was performed by washing the sectioned tissue three times in 100% xylene, twice in 100% ethanol, once in 95% ethanol, once in 70% ethanol, once in 50% ethanol, and once in DI water. Each wash step was performed for 5 minutes. Antigen retrieval was performed by incubating the tissue sections in 95°C, 20 mM sodium citrate (pH 7.6) buffer for 20 minutes. Samples were left in sodium citrate buffer until the temperature reduced to 40°C. Samples were then rehydrated with two quick (< 1 minute) rinses in DI water followed by one five-minute wash in TBS-T.

Prior to staining, tissue sections were blocked with blocking buffer (*Dako*) for one hour. Tissue sections were stained to identify Salmonella and released GFP with 1:100 dilutions of [1] FITC-conjugated rabbit anti-Salmonella polyclonal antibody (*Abcam*, catalog # ab69253), and [2] rat anti-*myc* monoclonal antibody (*Chromotek*) in Tris buffered saline with 0.1% Tween 20 (TBS-T) with 2% BSA (*FisherScientific*). Sections were washed three times in TBS-T w/2% BSA and incubated with Alexaflor-568 goat anti-rat secondary antibodies (*ThermoFisher*). After washing sections three times with TBS-T, 40 µl of mountant with DAPI (*ThermoFisher*) and a cover slip were added to each slide. Slides were incubated at room temperature for 24 hours until the mountant solidified. Slides were imaged as described in the *microscopy* section.

Delivery fraction in tumor sections was quantified using a similar method as with fixed cells on cover slips described above. Invaded cells were identified as nuclei bordering intracellular Salmonella and cells with delivered protein had GFP throughout the cytosol. The delivery fraction was the number of cells with delivered protein divided by the total number of infected cells. Image analysis was blinded and conducted without knowledge of the treatment group.

### CD8 T cell activation and culturing

To isolate OT-I CD8 T cells, the spleen and inguinal lymph nodes were harvested from female OT-I mice. The lymphoid tissue was mechanically dissociated in PBS using the end of a syringe. A single cell suspension was produced by passing the organ slurry through a 40 micrometer cell strainer. Naïve OT-I T cells were purified using a negative selection kit (*Biolegend*). This negative selection purified approximately eight to ten million naïve OT-I T cells, which were 91% pure.

The isolated T cells were activated using anti-CD3 and anti-CD28 antibodies and either (1) a plate-bound method or (2) magnetic beads (*Thermo-Fisher*). The plate-bound method was used to prepare T cells for addition to cancer cells in culture flasks. The magnetic bead method was used to prepare T cells for adoptive transfer into tumor-bearing mice. For both methods, one million purified, naïve OT-I T cells were added to 5 ml of complete RPMI media (2 mM glutamine, 2 mM sodium pyruvate, 20 IU/ml recombinant mouse IL-2, 50 µM beta-mercaptoethanol and 12.5 µg/ml amphotericin B in RPMI media).

To prepare the antibody plate, anti-CD3ϵ antibody (*Biolegend*) was added in 2 ml of PBS to a T25 flask at a concentration of 4 µg/ml and incubated at 37°C overnight. The flask was washed twice with 5 ml of PBS to remove unbound antibody. T cells were added to the treated flask and the medium was supplemented with 2 µg/ml of anti-CD28 antibody (*Biolegend*). The T cells were incubated for 4 days at 37°C. After 4 days in the activation media, the cells were washed three times with PBS to remove the CD3 and CD28 antibodies. The T cells were maintained at a concentration between 500,000-1,000,000 cells/ml.

For the bead method, 25 µl of washed CD3/CD28 Dynabeads were added to naïve T cells. After incubating at 37°C for 96 hours, cell clusters were gently broken apart by pipetting. A magnet was used to separate the magnetic beads from the activated T cells. The separated T cells were washed twice with PBS, re-suspended in complete RPMI medium and maintained at a concentration of 1 million cells/ml.

Five days after starting the activation process, the OT-I T cells were stained against CD8 and CD44 to assess purity and extent of activation, respectively. The anti-CD8 and anti-CD44 antibodies were conjugated to APC and FITC (*Biolegend*), respectively, and diluted 1:500 in extracellular staining buffer (ESB; PBS with 1 mM EDTA and 2% BSA). Stained samples were evaluated on a Novocyte flow cytometer. Fluorescence minus one and unstained T cells were used as gating controls.

### T cell cytotoxicity after ovalbumin delivery *in vitro*

To measure the effect of bacterial ovalbumin delivery on T cell-cytotoxicity, OT-I T cells were applied to cancer cells after being infected with antigen-delivering Salmonella. ID-GFP and ID-OVA were grown to an OD of 0.8 in LB. These bacteria were added to well-plates containing 60% confluent Hepa 1-6 cells at an MOI of 20 for two hours. The bacteria were induced with 20 mM arabinose during the 2-hour infection. After infection, the cancer cells were washed five times with PBS to eliminate extracellular bacteria. The cells were incubated in complete RPMI medium containing 50 µg/ml gentamycin and 1 µM calcein-AM for 30 minutes. The cells were washed three times with PBS to eliminate the extracellular calcein-AM. These treated Hepa 1-6 cells were incubated with isolated and activated OT-I CD8 T cells at an effector-to-target ratio of 10:1 in complete RPMI medium (50 µM beta-mercaptoethanol, 20 IU IL-2/ml, 2 mM sodium pyruvate, and 2 mM glutamine) for 48 hours. At the end of the incubation period, 200 µl of RPMI media was sampled from each of the wells. The 200 µl samples was centrifuged at 1000×g for 5 minutes. For each 100 µl sample, the fluorescence intensity from released calcein was quantified using a plate reader (*Biotek*).

### Efficacy of ovalbumin delivery in mice after T cell adoptive transfer

Two groups of six week-old C57BL/6 mice were subcutaneously injected with 1×10^5^ MC38 cancer cells. Once tumors reached approximately 50 mm^3^, the mice were intratumorally injected with 4x10^7^ GFP-delivering (ID-GFP) or ovalbumin-delivering (ID-OVA) Salmonella. Forty-eight hours days after bacterial injection (designated as day 0), one million activated, OT-I T cells were adoptively transferred into each mouse through the tail vein. In addition, 48 and 72 hours after bacterial injection, the mice were injected (IP) with 100 mg of arabinose in 400 µl of PBS to induce *flhDC* expression. The bacteria and T cell administration cycle was performed twice for each mouse. Tumor volumes were measured with a caliper twice a week until they reached maximum volume limits or cleared. Tumor volumes were calculated using the formula (Length)x(Width)^2^/2.

The effect of ovalbumin delivery in the absence of adoptive transfer was measured in two groups of female mice that were subcutaneously injected with 1x10^5^ MC38 cells. Once tumors were approximately 50 mm^3^, mice were intratumorally injected with 4x10^6^ CFU of ID-GFP or ID-OVA every four days. The first bacterial injection (day 1) was one day after the first tumor measurement. One hundred milligrams of arabinose were injected IP into the mice at 48 and 72 hours after bacterial injection. Tumors were measured with calipers every 3 days until mice reached maximal tumor burden.

### Delivery and efficacy of ovalbumin delivery *in vivo* after immunization

Two groups of six-week-old female C57BL/6 mice were immunized by two IP injections of 100 µg ovalbumin and 100 µg poly(I:C) in 100 µl PBS spaced seven days apart. Fourteen days after the immunization booster, the mice were subcutaneously injected with 1x10^5^ MC38 cancer cells on the hind flank. Once the tumors reached approximately 50 mm^3^, the mice were intratumorally injected with 4x10^7^ of either GFP-delivering (ID-GFP) or ovalbumin-delivering (ID-OVA) Salmonella. The first bacterial injection (day 1) was one day after the first tumor measurement. Forty-eight hours after bacterial injection, the mice were injected (IP) with 50 µg of anti-PD-1 checkpoint blockade antibodies (*Biolegend*). In addition, 48 and 72 hours after bacterial injection, mice were injected IP with 100 µg arabinose. The treatment cycle was performed twice for each mouse. Tumor volumes were measured with calipers twice a week until they reached maximum volume limits. Tumor volumes were calculated using the formula (Length)x(Width)^2^/2.

### Treatment of immunized mice with ID-OVA and tumor re-challenge

Four groups of female C57BL/6 mice were immunized with 100 µg ovalbumin and 50 µg poly(I:C) in 100 µl PBS by IP injection, 28 days apart. One week after the second immunization, the mice were subcutaneously injected with 2×10^5^ KPC PDAC cells (*Kerafast*) on the right flank. Once tumors reached approximately 30-50 mm^3^, the mice were injected intratumorally with either 1×10^7^ CFU of ID-OVA, 1×10^7^ CFU of ID-GFP (bacterial control), saline, or intraperitoneally injected with 50 mg/kg gemcitabine every 5 days. All mice were injected (IP) with 400 mg of arabinose 48 and 72 hours after therapeutic administration. Tumors were measured using calipers every three days. Tumor volumes were calculated using the formula (length*width^2^)/2. Mice that completely cleared tumors were re-challenged on the left flank 14 days after primary tumor clearance and monitored for tumor regrowth for a minimum of 14 days.

## Data availability statement

The raw data supporting the conclusions of this article will be made available by the authors, without undue reservation.

## Ethics statement

Ethical approval was not required for the studies on humans in accordance with the local legislation and institutional requirements because only commercially available established cell lines were used. The animal study was approved by UMass Institutional Animal Care and Use Committee (IACUC). The study was conducted in accordance with the local legislation and institutional requirements.

## Author contributions

VR and NF contributed to conception and design of the study. VR, LH, SB, CH, and VW performed experiments. VR, CH, and NF interpreted data and performed statistical analysis. VR wrote the first draft of the manuscript. VR, LM, AK, and NF wrote and edited the manuscript. All authors contributed to manuscript revision, read, and approved the submitted version.
